# Trends in incidence and mortality of lung cancer and age-period-cohort model analysis in Zibo, Shandong Province, 2014–2024

**DOI:** 10.3389/fonc.2026.1873592

**Published:** 2026-07-28

**Authors:** Jingjing Liu, Xuan Zhao, Shuxia Yang, Linlin Li

**Affiliations:** 1 Institute of Endemic and Chronic Disease Control, Zibo Center for Disease Control and Prevention, Zibo, Shandong, China; 2 School of Public Health, Shandong University-Joint Department of Environment and Health, Zibo Center for Disease Control and Prevention, Zibo, Shandong, China

**Keywords:** age-period-cohort model, incidence, lung cancer, mortality, trends

## Abstract

**Background:**

Lung cancer is the leading malignancy worldwide and a major public health challenge in China. Driven by population aging, tobacco use and air pollution, its disease burden remains severe. Zibo is a densely populated industrial city with a heavy lung cancer burden. Understanding the incidence and mortality trends and quantifying age-period-cohort effects are critical for targeted prevention and control. This study analyzed data from 2014 to 2024 to identify trends and high-risk populations to inform evidence-based intervention strategies.

**Objective:**

This study aimed to examine trends in lung cancer incidence and mortality among residents of Zibo from 2014 to 2024, evaluate the independent effects of age, period and birth cohort, identify high-risk populations and thereby provide evidence for developing targeted lung cancer prevention and intervention strategies.

**Methods:**

Individual registration data on lung cancer incidence and mortality among registered residents in Zibo from 2014 to 2024 were obtained. The joinpoint regression was used to analyze temporal trends in incidence and mortality rates, and the average annual percentage change (AAPC) with 95% confidence interval (*CI*) was calculated. The age-period-cohort model was applied to quantify the effects of age, period and cohort on lung cancer incidence and mortality risk among residents aged ≥20 years.

**Results:**

From 2014 to 2024, the age-standardized incidence rates of lung cancer among males, females and the overall population in Zibo all showed a significant upward trend, while the age-standardized mortality rates showed a downward trend. The AAPC for the overall standardized incidence rate was 4.34% (95% *CI*: 2.14–6.59, *p<* 0.05), and the AAPC for the overall standardized mortality rate was -2.13% (95% *CI*: -3.53 to -0.72, *p<* 0.05). The standardized incidence and mortality rates among males were consistently higher than those in females (*p<* 0.05 for both). The age effect on lung cancer incidence first increased and then decreased with age, the period effect increased over time, and the cohort effect decreased with successive birth cohorts. The age effect on lung cancer mortality increased with age, the period effect increased over time among male, and decreased over time among females. The cohort effect also generally decreased across successive birth cohorts.

**Conclusion:**

From 2014 to 2024, the incidence rate of lung cancer in Zibo continued to increase, while the mortality rate showed a downward trend. Prevention and control of lung cancer should be continuously strengthened in the elderly and males.

## 1 Introduction

Lung cancer is one of the most common cancers in China and continues to pose a severe and persistent threat to the life and health of the population. Epidemiological data from the past decade showed that both lung cancer incidence and mortality rates in China have continued to rise (1, 2). According to the GLOBOCAN 2022 estimates, approximately 2,481,000 new lung cancer cases were diagnosed worldwide in 2022, accounting for 12.4% of all new cancer cases and ranking first among all cancers. Lung cancer also caused an estimated 1,817,000 deaths, representing 18.7% of all cancer-related deaths, making it the leading cause of cancer mortality globally. China has the highest lung cancer burden worldwide. In 2022, China reported about 1,061,000 new cases and 733,000 lung cancer deaths, accounting for 42.8% and 40.4% of the global totals, respectively—far exceeding any other country (3). Among all cancers in Zibo, lung cancer ranks first in both incidence and mortality (4, 5), and the associated public health burden has continued to increase.

By 2020, the proportion of residents aged ≥60 years in Zibo had reached 23.24%, indicating a markedly aged population. As an old industrial city, Zibo faces the superimposed challenges of population aging and legacy industrial pollution. Against this backdrop, this study aimed to examine the epidemiological trends and population distribution of lung cancer incidence and mortality in Zibo from 2014 to 2024. Joinpoint regression and Age-Period-Cohort models were used to analyze temporal trends and quantify the independent effects of age, period, and birth cohort on lung cancer risk. The findings aim to provide scientific evidence for local lung cancer prevention and control strategies.

## 2 Materials and methods

### 2.1 Data sources

Lung cancer incidence and mortality data for Zibo from 2014 to 2024 were obtained from the Shandong Provincial Comprehensive Management Information System for Chronic Disease and Mortality Surveillance System. Cases were extracted in accordance with the classification rules of the International Classification of Diseases, 10th Revision (ICD-10), and those coded as trachea, bronchus, and lung cancer (C33-C34) were included. Annual registered population data were obtained from the Zibo Public Security Bureau.

### 2.2 Quality control

Data quality was assessed based on the Guidelines for Chinese Cancer Registration and the standards set by the International Agency for Research on Cancer (IARC) and the International Association of Cancer Registries (IACR). From 2014 to 2024, the morphological verification percentage (MV%) of cancer registration data in Zibo was 75.98%, the percentage of cases registered by death certificate only (DCO%) was 4.12%, and the mortality-to-incidence ratio (MI) was 0.72. All quality indicators met the national and international standards, indicating that the lung cancer registry data in Zibo were reliable, complete, and valid.

### 2.3 Methods

#### 2.3.1 Joinpoint trend analysis

Joinpoint regression was used to analyze temporal trends in the age-standardized incidence and mortality rates of lung cancer. An incidence and mortality data generally follow a Poisson distribution, a log-linear model was used (6). Model parameters were set as follows: based on a total of 11 annual data points from 2014 to 2024, the maximum number of joinpoints was set to 2 to ensure that each segment contained at least four observations for stable estimation. The grid search method identified candidate joinpoint locations, and the optimal number was determined by the Monte Carlo permutation test with 4,499 permutations and a significance level of α = 0.05 (7, 8). Model selection was based on the permutation test, which sequentially compares k joinpoints versus k + 1 joinpoints. Error terms were assumed to be independent across years (7). The annual percentage change (APC) and average annual percentage change (AAPC) with their 95% confidence intervals (*CI*) were calculated for each segment and the entire period, respectively. A positive APC/AAPC indicates an upward trend, while a negative value indicates a downward trend (9). When no joinpoints are identified, APC = AAPC, indicating a monotonic trend over the entire period.

#### 2.3.2 Age-period-cohort model analysis

The age-period-cohort model is based on the Poisson distribution and simultaneously estimates the effects of age, period, and cohort on the disease risk simultaneously. Because of the perfect linear dependency among age, period, and cohort (period = age + cohort), conventional regression models cannot produce a unique parameter solution. To solve this, the intrinsic estimator (IE) method was adopted for model estimation (10). The IE method imposes sum-to-zero and minimum variance constraints to obtain a unique solution, in which each effect coefficient represents the deviation of that specific group from the overall mean across all groups: a coefficient< 0 indicates a decreased risk, a coefficient > 0 indicates an increased risk, with higher values indicating a higher risk. Relative risks (RR) for each group were then calculated by exponentiating the coefficients (RR = exp (coefficient)). Model goodness-of-fit was comprehensively evaluated using the Akaike information criterion (AIC), Bayesian information criterion (BIC), deviance, and log-likelihood ratio (11, 12). The basic expression of the age-period-cohort model is:


$$\text{Y}=\mu +\alpha_{\text{Age}}+\beta_{\text{Period}}+\gamma_{\text{Cohort}}+\epsilon$$

where *Y* denotes the lung cancer incidence or mortality rate, *μ* is the intercept term, *α*, *β*, and *γ* represent the age effect, period effect, and birth cohort effect, respectively, and *ϵ* denotes the residual term.

Given the low incidence and mortality rates of lung cancer among individuals under 20 years, the study population was restricted to those ≥20 years to ensure the validity of the analysis. Data were stratified into 14 five-year age groups (20~, 25~, 30~, 35~, 40~, 45~, 50~, 55~, 60~, 65~, 70~, 75~, 80~, and ≥85). To avoid broadening the birth cohort range and thus compromising the temporal precision of the risk description, age-specific data from the years 2014, 2019, and 2024 were selected for the age-period-cohort model simulation (13). Although the study period was only 11 years, the 14 age groups covered a birth cohort span of over 60 years, providing enough information to estimate cohort effects. Using a three-period categories age-period-cohort model has precedent in cancer epidemiology (14) and supports the interpretation of trends.

### 2.4 Statistical methods

Data management, statistical analysis, and figure generation were performed using SAS 9.4, Excel 2016, and GraphPad Prism 12. Age-standardized incidence rates were calculated using the 2000 Chinese standard population. Standardized rates for males and females were compared using rate ratios and *CI*. Joinpoint regression was performed using the Joinpoint Regression Program 4.9.1.0 (National Cancer Institute, USA). The age-period-cohort analysis was conducted using the APC-IE package in Stata 18.0.3. The significance level was set at *α* = 0.05.

## 3 Results

### 3.1 Incidence and mortality of lung cancer

From 2014 to 2024, 42,284 new lung cancer cases were reported in Zibo, including 25,143 among males and 17,141 among females. The crude incidence rate was 88.94 per 100,000, and the age-standardized incidence rate was 42.60 per 100,000. The age-standardized incidence rate was 49.62 per 100,000 among males and 35.51 per 100,000 in females, with a male−to−female rate ratio of 1.40 (95% *CI*: 1.37–1.43, *p* < 0.05) (**Table 1**).

**Table 1:** Crude and age-standardized incidence rates of lung cancer in Zibo, 2014–2024 (/10^5^).

Year	Both			Male			Female		
	Cases	Crude rate	Age-standardized rate	Cases	Crude rate	Age-standardized rate	Cases	Crude rate	Age-standardized rate
2014	2828	65.84	33.23	1770	82.70	43.47	1058	49.09	23.83
2015	3074	71.56	35.82	1971	92.09	48.05	1103	51.18	24.63
2016	2995	69.16	35.56	1896	87.90	47.34	1099	50.56	25.00
2017	3125	72.16	37.03	1912	88.65	47.35	1213	55.80	27.77
2018	3345	77.24	39.49	2076	96.25	51.13	1269	58.38	29.21
2019	3676	84.69	41.08	2215	102.74	51.97	1461	66.87	31.48
2020	4661	107.38	52.44	2715	125.94	63.43	1946	89.07	43.10
2021	4849	111.90	52.52	2786	129.58	61.01	2063	94.49	45.22
2022	4462	102.97	47.54	2546	118.42	54.73	1916	87.76	41.52
2023	4521	105.03	45.71	2565	120.43	52.29	1956	89.95	40.26
2024	4748	110.31	47.50	2691	126.35	54.49	2057	94.59	41.77
Total	42284	88.94	42.60	25143	106.44	49.62	17141	71.67	35.51
AAPC, % (95% *CI*)	–	–	4.34 (2.14, 6.59)	–	–	2.53 (0.54, 4.56)	–	–	6.58 (2.37, 10.97)
P-value	–	–	< 0.05	–	–	< 0.05	–	–	< 0.05

During this period, 30,421 lung cancer deaths were reported in Zibo, including 19,846 among males and 10,575 among females. The crude mortality rate was 63.99 per 100,000, and the age−standardized mortality was 28.03 per 100,000. The age−standardized mortality rate was 36.45 per 100,000 among males and 19.46 per 100,000 among females, with a male−to−female rate ratio of 1.87 (95% *CI*: 1.83–1.92, *p* < 0.05) (**Table 2**).

**Table 2:** Crude and age-standardized mortality rates of lung cancer in Zibo, 2014–2024 (/10^5^).

Year	Both			Male			Female		
	Cases	Crude rate	Age-standardized rate	Cases	Crude rate	Age-standardized rate	Cases	Crude rate	Age-standardized rate
2014	2548	59.32	28.51	1611	75.27	38.12	937	43.48	19.91
2015	2734	63.65	30.52	1792	83.73	42.51	942	43.71	19.74
2016	2720	62.81	30.74	1774	82.25	42.98	946	43.52	19.83
2017	2744	63.36	30.75	1788	82.90	43.40	956	43.98	19.38
2018	2772	64.01	30.50	1792	83.08	42.77	980	45.08	19.62
2019	2803	64.58	29.29	1849	85.77	41.80	954	43.67	18.46
2020	2933	67.57	30.14	1920	89.06	43.14	1013	46.37	18.93
2021	2789	64.36	25.79	1803	83.86	36.17	986	45.16	16.72
2022	2790	64.38	25.94	1839	85.53	37.24	951	43.56	15.89
2023	2660	61.80	23.21	1748	82.07	33.30	912	41.94	14.49
2024	2928	68.02	25.35	1930	90.62	36.49	998	45.89	15.63
Total	30421	63.99	28.03	19846	84.01	36.45	10575	44.22	19.46
AAPC, % (95% *CI*)	–	–	-2.13 (-3.53, -0.72)	–	–	-1.65 (-3.27, 0.00)	–	–	-2.89 (-3.97, -1.80)
P-value	–	–	< 0.05	–	–	> 0.05	–	–	< 0.05

### 3.2 Age-specific characteristics and heatmap distribution

**Figure 1:**
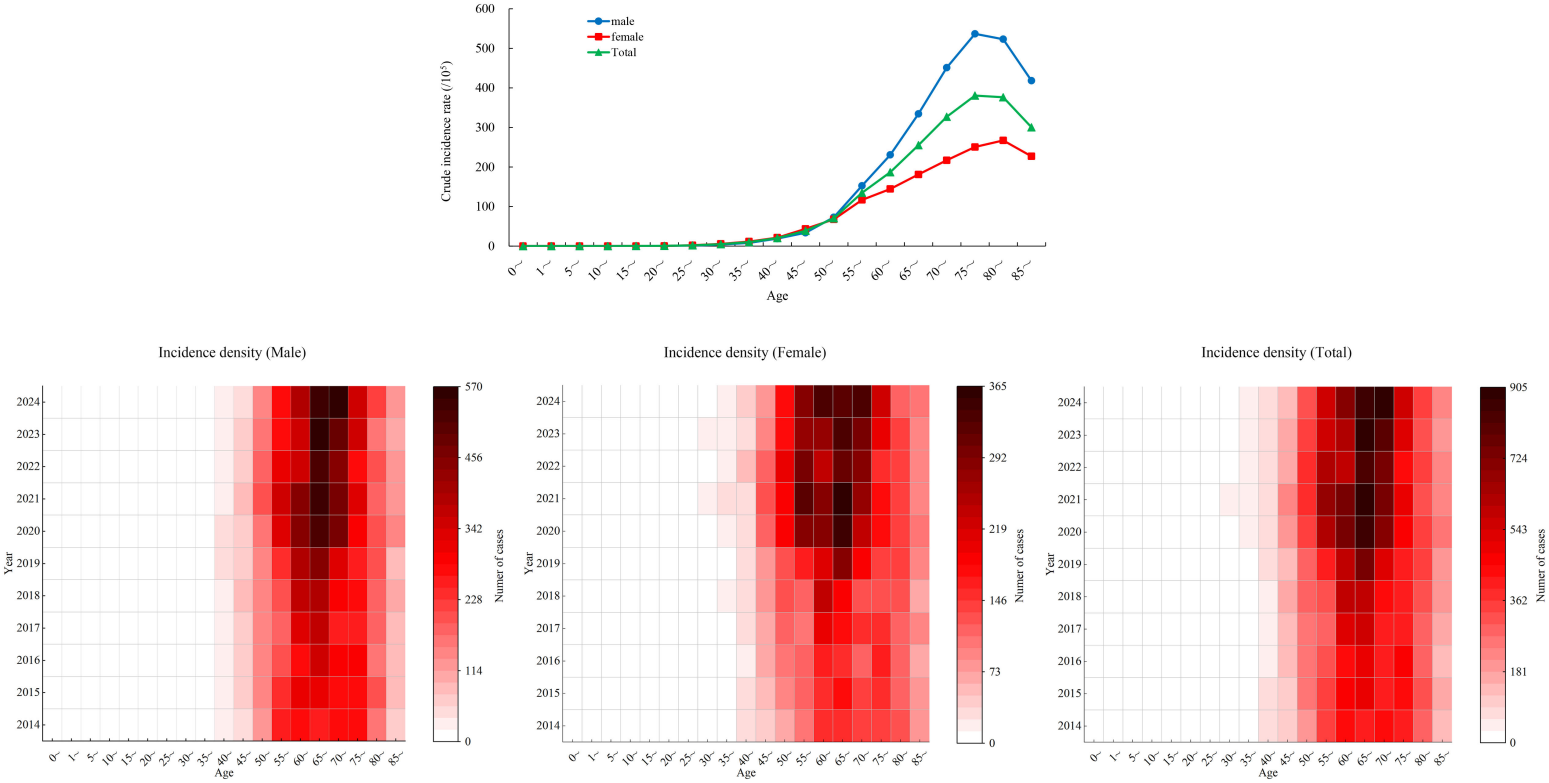
Age-specific incidence rates and case density of lung cancer in Zibo, 2014–2024.

**Figure 1** shows the age-specific incidence rates and case density for lung cancer in Zibo from 2014 to 2024. Overall, lung cancer incidence increased with age, particularly after age 45, and peaked after age 80. Incidence rates for both males and females also increased with age, consistent with the overall trend. The heatmap revealed that, over time, the age groups with the highest incidence gradually shifted toward 65–69 years for the total population, 65–74 years for males, and 55–74 years for females from 2014 to 2024.

**Figure 2:**
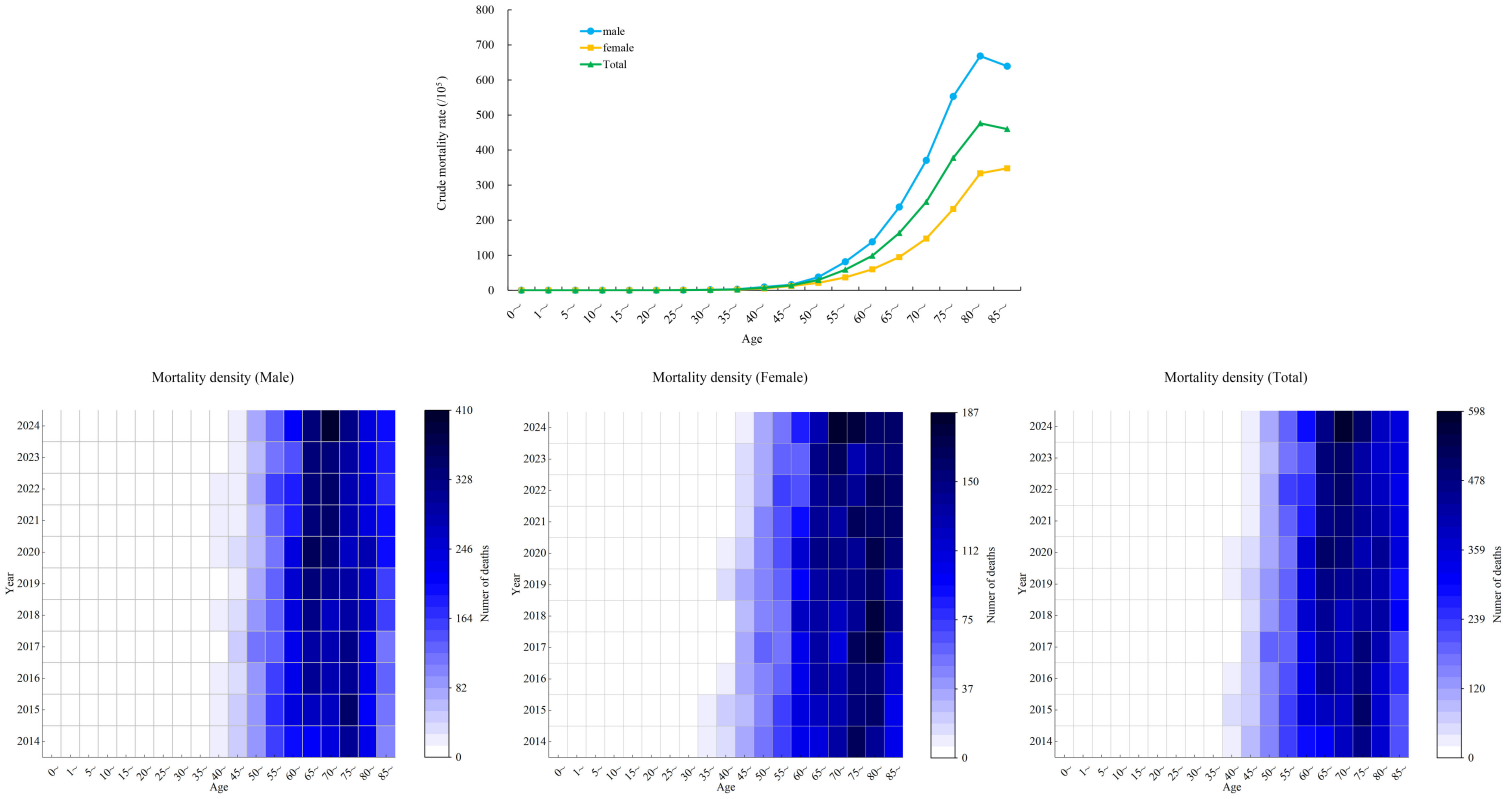
Age-specific mortality rates and case density of lung cancer in Zibo, 2014–2024.

**Figure 2** shows the age-specific mortality rates and case density for lung cancer in Zibo from 2014 to 2024. Overall, lung cancer mortality increased with age, rising sharply after age 55 and peaking around age 80. Mortality patterns for both males and females were consistent with the overall trend. The heatmap showed that, over time, the age groups with the highest mortality gradually shifted toward 65–79 years for the total population, 65–79 years for males, and 70–84 years for females from 2014 to 2024.

### 3.3 Trends in lung cancer incidence

**Figure 3:**
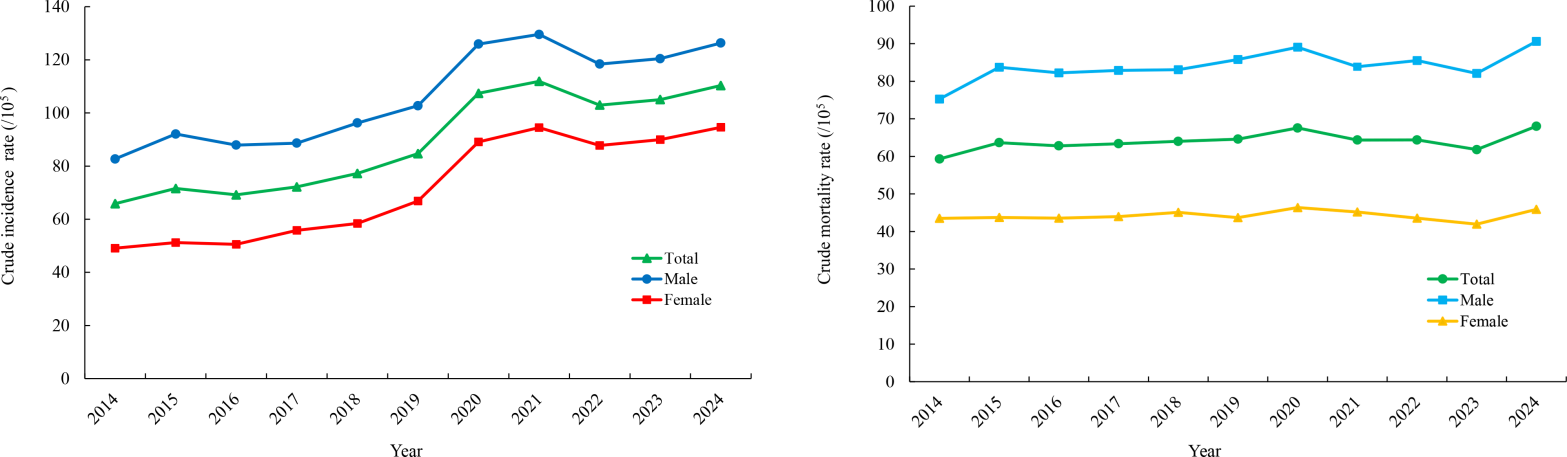
Incidence and mortality trends of lung cancer by gender in Zibo, 2014–2024.

From 2014 to 2024, the crude incidence rate of lung cancer in Zibo showed a continuous upward trend, increasing from 65.84 per 100,000 in 2014 to 110.10 per 100,000 in 2024. Rates among males remained consistently higher than those among females, and the trends for both sexes were consistent with the overall trend (**Figure 3**).

**Figure 4:**
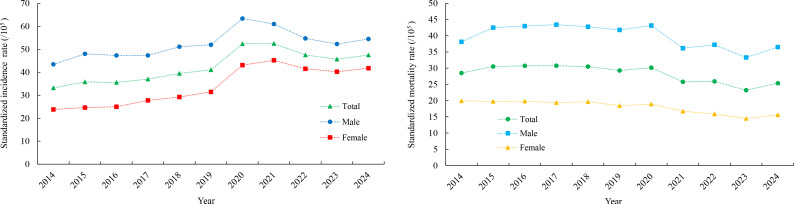
Trends in age-standardised incidence and mortality of lung cancer by gender in Zibo, 2014–2024.

**Figure 4** shows an upward trend in the age-standardized incidence rate of lung cancer in Zibo, rising from 33.23 per 100,000 in 2014 to 47.50 per 100,000 in 2024, with a sharp rise after 2019. Throughout the study period, the rate remained consistently higher among males than among females. Among females, the age-standardized incidence rate increased steadily from 2014 to 2021 and plateaued thereafter. Joinpoint regression analysis (**Table 1**) revealed a significant increase in the overall age-standardized incidence rate of lung cancer over the entire study period (AAPC = 4.34%, 95% *CI*: 2.14–6.59, *p* < 0.05). A similar upward trend was observed among males (AAPC = 2.53%, 95% *CI*: 0.54–4.56, *p* < 0.05) and females (AAPC = 6.58%, 95% *CI*: 2.37–10.97, *p* < 0.05).

### 3.4 Trends in lung cancer mortality

From 2014 to 2024, the crude mortality rate of lung cancer in Zibo remained relatively stable. The crude mortality rate among males was higher than the city average and substantially higher than that among females, and the temporal trends for both sexes were consistent with the overall trend (**Figure 3**).

**Figure 4** presents the temporal trend in the age-standardized mortality rate of lung cancer in Zibo from 2014 to 2024. The overall age-standardized mortality rate declined from 28.51 per 100,000 in 2014 to 25.35 per 100,000 in 2024, with the most pronounced decline observed after 2020. Throughout the study period, this rate was consistently higher among males than females. This downward trend was considerably more pronounced in females, whereas males exhibited only a modest decline. Joinpoint regression analysis (**Table 2**) revealed a significant overall decline in the age-standardized mortality rate (AAPC = -2.13%, 95% *CI*: -3.53 to -0.72, *p* < 0.05). By sex, a significant decline was observed among females (AAPC = -2.89%, 95% *CI*: -3.97 to -1.80, *p* < 0.05), whereas the decrease in males did not reach statistical significance (AAPC = -1.65%, 95% *CI*: -3.27 to 0.00, *p* > 0.05).

### 3.5 Age-period-cohort model analysis

**Figure 5:**
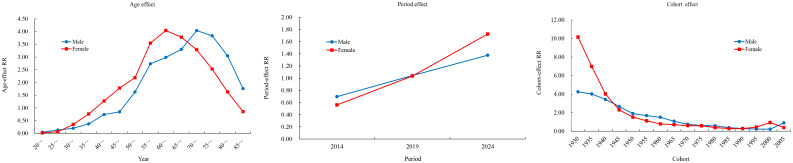
Relative risk from the age-period-cohort model for lung cancer incidence in Zibo.

**Table 3** and **Figure 5** show the independent effects of age, period, and birth cohort on lung cancer incidence risk in the age-period-cohort model. Among those aged ≥20 years, lung cancer incidence risk first increased and then decreased with advancing age, but the turning points differed by sex. The lowest risk was in the 20–24 age group, with relative risk (RR) values of 0.045 for males and 0.019 for females. For males, the risk rose sharply after age 50, peaked in the 70–74 age group (RR = 4.041), and then declined slightly but remained elevated. For females, the risk increased rapidly after age 50, peaked earlier at age 60–64 (RR = 4.043), and gradually decreased thereafter. Period effects showed that lung cancer incidence risk increased over time in both sexes, with a steeper increase observed in females than in males. Cohort effects showed that, lung cancer incidence risk declined progressively across successive birth cohorts in both sexes. In early birth cohorts, females showed a stronger cohort effect than males and a more pronounced downward trend.

**Table 3:** Age-period-cohort effect analysis for lung cancer incidence in Zibo.

Groups	Male				Female			
	Coefficient	Standard error	*Z*	*P*	Coefficient	Standard error	*Z*	*P*
20∼	-3.106	0.956	-3.250	0.001	-3.979	0.986	-4.030	<0.001
25∼	-2.032	0.480	-4.240	<0.001	-2.748	0.578	-4.750	<0.001
30∼	-1.605	0.378	-4.250	<0.001	-1.041	0.379	-2.750	0.006
35∼	-0.987	0.318	-3.100	0.002	-0.272	0.338	-0.810	0.420
40∼	-0.300	0.278	-1.080	0.280	0.242	0.298	0.810	0.417
45∼	-0.169	0.242	-0.700	0.487	0.576	0.257	2.240	0.025
50∼	0.486	0.198	2.460	0.014	0.784	0.215	3.650	<0.001
55∼	1.005	0.153	6.580	<0.001	1.266	0.170	7.440	<0.001
60∼	1.094	0.110	9.900	<0.001	1.397	0.126	11.060	<0.001
65∼	1.194	0.074	16.160	<0.001	1.330	0.087	15.300	<0.001
70∼	1.396	0.058	24.210	<0.001	1.189	0.063	19.000	<0.001
75∼	1.344	0.078	17.200	<0.001	0.928	0.074	12.460	<0.001
80∼	1.114	0.118	9.470	<0.001	0.487	0.113	4.320	<0.001
85∼	0.564	0.164	3.440	0.001	-0.158	0.161	-0.980	0.328
Period (Year)								
2014	-0.361	0.051	-7.150	<0.001	-0.580	0.054	-10.650	<0.001
2019	0.041	0.016	2.570	0.010	0.035	0.022	1.590	0.112
2024	0.320	0.050	6.450	<0.001	0.545	0.052	10.510	<0.001
Cohort (Year)								
1924-1929	1.447	0.200	7.240	<0.001	2.318	0.231	10.050	<0.001
1929-1934	1.392	0.147	9.440	<0.001	1.944	0.178	10.900	<0.001
1934-1939	1.231	0.102	12.080	<0.001	1.389	0.137	10.160	<0.001
1939-1944	0.981	0.066	14.870	<0.001	0.832	0.108	7.690	<0.001
1944-1949	0.640	0.058	11.030	<0.001	0.415	0.101	4.120	<0.001
1949-1954	0.521	0.084	6.180	<0.001	0.107	0.118	0.910	0.362
1954-1959	0.405	0.125	3.240	0.001	-0.248	0.150	-1.650	0.099
1959-1964	0.067	0.169	0.400	0.692	-0.354	0.189	-1.870	0.061
1964-1969	-0.294	0.215	-1.370	0.171	-0.512	0.230	-2.220	0.026
1969-1974	-0.491	0.258	-1.900	0.057	-0.550	0.271	-2.030	0.042
1974-1979	-0.554	0.297	-1.870	0.062	-0.958	0.311	-3.080	0.002
1979-1984	-0.981	0.335	-2.930	0.003	-1.261	0.348	-3.620	<0.001
1984-1989	-1.244	0.370	-3.360	0.001	-1.280	0.384	-3.330	0.001
1989-1994	-1.473	0.450	-3.270	0.001	-0.829	0.422	-1.970	0.049
1994-1999	-1.533	0.641	-2.390	0.017	-0.071	0.637	-0.110	0.911
1999-2004	-0.113	1.075	-0.110	0.916	-0.945	1.619	-0.580	0.559
AIC	7.83				7.39			
BIC	-17.32				-24.71			
Deviance	2.29				1.68			
Log likelihood	-134.38				-125.10			

**Figure 6:**
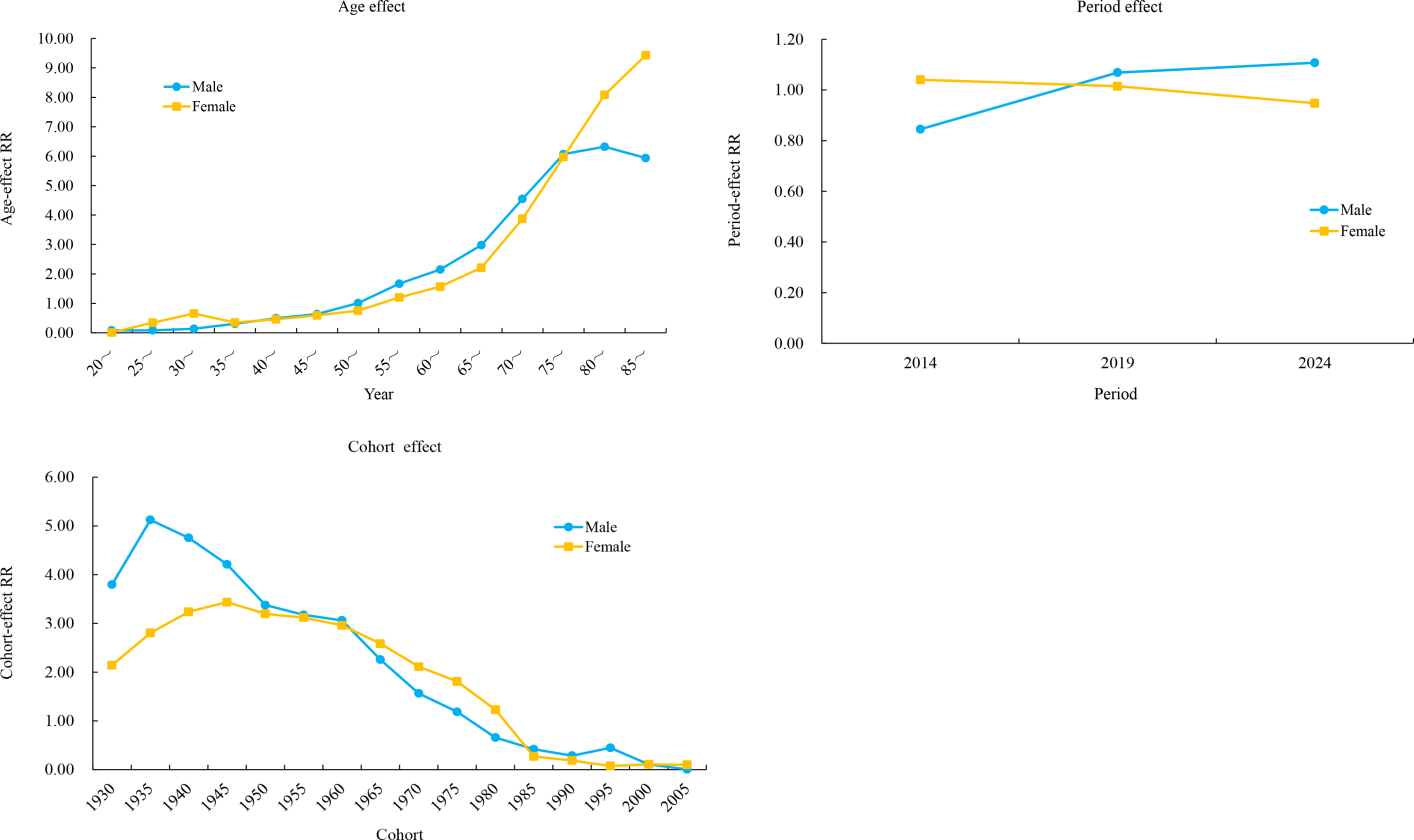
Relative risk from the age-period-cohort model for lung cancer mortality in Zibo.

**Table 4** and **Figure 6** show the effects of age, period, and birth cohort on lung cancer mortality risk in the age-period-cohort model. Age effects showed that, the lung cancer mortality risk increased steadily with age in both sexes, with a greater increase in females. The lowest risk was in the 20–24 age group, with RR values of 0.082 for males and 0.008 for females. For males, mortality risk rose more steeply after age 55, peaked at age 80–84 (RR = 6.322), and then declined slightly. For females, mortality risk was relatively stable from age 20 to 50, increased sharply after age 65, and peaked in the ≥85 age group (RR = 9.430). Period effects showed that, the lung cancer mortality risk increased over time in males, but decreased gradually in females. Cohort effects showed that mortality risk declined across successive birth cohorts. Compared with the latest birth cohort (1999–2004), those in the earliest cohort (1924–1929) had a 21.50-fold higher mortality risk in males and a 36.40-fold higher risk in females. In early birth cohorts, the cohort effect was stronger among males, who also showed a greater decline than females.

**Table 4:** Age-period-cohort effect analysis for lung cancer mortality in Zibo.

Groups	Male				Female			
	Coefficient	Standard error	*Z*	*P*	Coefficient	Standard error	*Z*	*P*
20∼	-2.497	1.010	-2.470	0.013	-4.775	5.980	-0.800	0.425
25∼	-2.490	0.785	-3.170	0.002	-1.063	1.305	-0.810	0.415
30∼	-1.988	0.564	-3.520	<0.001	-0.422	1.116	-0.380	0.705
35∼	-1.188	0.491	-2.420	0.016	-1.051	1.006	-1.040	0.296
40∼	-0.701	0.421	-1.670	0.096	-0.784	0.881	-0.890	0.374
45∼	-0.454	0.357	-1.270	0.204	-0.523	0.757	-0.690	0.490
50∼	0.007	0.285	0.030	0.979	-0.281	0.636	-0.440	0.658
55∼	0.511	0.215	2.380	0.017	0.182	0.512	0.360	0.722
60∼	0.766	0.151	5.060	<0.001	0.450	0.392	1.150	0.252
65∼	1.092	0.105	10.440	<0.001	0.793	0.281	2.820	0.005
70∼	1.514	0.105	14.490	<0.001	1.354	0.196	6.900	<0.001
75∼	1.803	0.152	11.860	<0.001	1.787	0.182	9.840	<0.001
80∼	1.844	0.218	8.460	<0.001	2.090	0.250	8.350	<0.001
85∼	1.781	0.290	6.130	<0.001	2.244	0.357	6.280	<0.001
Period (Year)								
2014	-0.168	0.078	-2.150	0.032	0.040	0.133	0.300	0.766
2019	0.066	0.016	4.200	<0.001	0.014	0.024	0.610	0.544
2024	0.102	0.078	1.300	0.192	-0.054	0.133	-0.410	0.685
Cohort (Year)								
1924-1929	1.334	0.626	2.130	0.033	0.762	0.705	1.080	0.279
1929-1934	1.634	0.603	2.710	0.007	1.031	0.640	1.610	0.107
1934-1939	1.559	0.589	2.650	0.008	1.175	0.599	1.960	0.050
1939-1944	1.438	0.586	2.450	0.014	1.234	0.586	2.100	0.035
1944-1949	1.217	0.593	2.050	0.040	1.162	0.603	1.930	0.054
1949-1954	1.155	0.610	1.890	0.058	1.138	0.648	1.760	0.079
1954-1959	1.119	0.636	1.760	0.079	1.085	0.713	1.520	0.128
1959-1964	0.815	0.670	1.220	0.224	0.950	0.795	1.190	0.232
1964-1969	0.447	0.711	0.630	0.529	0.748	0.887	0.840	0.399
1969-1974	0.171	0.755	0.230	0.821	0.594	0.986	0.600	0.547
1974-1979	-0.417	0.803	-0.520	0.603	0.207	1.086	0.190	0.849
1979-1984	-0.870	0.847	-1.030	0.305	-1.299	1.196	-1.090	0.278
1984-1989	-1.250	0.913	-1.370	0.171	-1.680	1.281	-1.310	0.190
1989-1994	-0.805	0.925	-0.870	0.385	-2.584	1.522	-1.700	0.090
1994-1999	-2.219	1.540	-1.440	0.150	-2.218	1.828	-1.210	0.225
1999-2004	-5.328	9.347	-0.570	0.569	-2.306	11.028	-0.210	0.834
AIC	7.25				6.36			
BIC	-20.69				-34.63			
Deviance	2.01				0.85			
Log likelihood	-122.32				-103.65			

## 4 Discussion

This study examined the epidemiological characteristics of lung cancer in Zibo over the past decade. The results showed that the age-standardized incidence and mortality rates in Zibo were 42.60 per 100,000 and 28.03 per 100,000, respectively, which were higher than the national averages (36.46 and 27.22 per 100,000) (15) and those in Jiangxi Province (35.97 and 27.35 per 100,000) (16), but lower than those in Heilongjiang (167.10 and 101.22 per 100,000) (17) and Hubei (184.14 and 96.91 per 100,000) (18). Compared with the provincial average (42.56 and 29.77 per 100,000) (19), the age-standardized incidence rate in Zibo was almost the same, while the age-standardized mortality rate was relatively lower.

The divergence between rising incidence and declining mortality may be partly explained by the interplay of several factors. First, population ageing has substantially intensified the lung cancer burden in Zibo: with 23.24% of residents aged ≥60 years, older adults are a high-risk population for lung cancer, and their risk rises steeply with advancing age (4). Second, the implementation of opportunistic cancer screening programs in Zibo, combined with the expanded use of low-dose spiral CT screening, has likely facilitated earlier detection at more treatable stages, thereby contributing to the observed increase in incidence. Third, the widespread adoption of targeted therapies and advances in multidisciplinary care have improved survival outcomes. Together with socioeconomic development and the enforcement of tobacco control policies (20), these factors may collectively account for the mortality decline despite rising incidence.

This study found that lung cancer mortality among males in Zibo was higher than that among females, which is consistent with the results of national studies (19, 21). This may be largely due to greater exposure among men to behavioral risk factors such as smoking and alcohol consumption (22, 23). Smoking is the primary risk factor for lung cancer. A local survey of 1,162 residents aged ≥15 years in Zibo reported a smoking prevalence of 21%, with a markedly higher rate in males than in females (24). The rate exceeded the provincial average of 20% among residents aged ≥15 years in Shandong Province (25). Tobacco contains nicotine, tar, carbon monoxide, and other harmful substances. Long-term smoking damages the respiratory mucosa, trachea, and alveolar tissue, promoting carcinogenesis (26). Studies have shown that lung cancer risk among smokers is much higher than that among nonsmokers, and the risk increases with the duration and intensity of smoking (26). A survey of chronic disease risk factors among adult residents in Zibo also found that the prevalence of smoking, regular drinking and overweight or obesity, and other behavioral risk factors was higher in males than in females (27). Men were also more frequently exposed to occupational hazards, such as dust and radioactive substances. Collectively, these behavioral and occupational exposures contribute to the persistently higher lung cancer burden in males and underscore the need for sex-specific prevention strategies. Strengthening tobacco control through increased taxation, comprehensive smoke-free legislation, and targeted health education—particularly for male residents—should remain a public health priority. At the same time, promoting healthy lifestyles (including reducing alcohol consumption, increasing physical activity, and minimizing occupational exposures) may help reduce the excess lung cancer risk in men. Notably, from 2014 to 2024, the increase in the age-standardized incidence rate was faster in females (AAPC = 6.58%) than in males (AAPC = 2.53%). This faster rise, despite the low smoking prevalence among women in Zibo, suggests that non-smoking-related risk factors play a substantial role. Potential contributors include greater exposure to second-hand smoke at home, cooking oil fumes during food preparation, and outdoor air pollution (28–30). The widening burden in women highlights the importance of expanding lung cancer prevention efforts beyond tobacco control to address indoor air quality and environmental exposures that disproportionately affect females.

Beyond behavioral factors, the environmental context of Zibo plays a critical role in shaping lung cancer risk. As a typical old industrial base, Zibo has a long history of petrochemical, metallurgical, coal, and building material industries, with high-intensity emissions leading to persistently elevated levels of PM_2.5_, polycyclic aromatic hydrocarbons (PAHs), and benzo[a]pyrene (BaP) in the atmosphere (31). The current trends in lung cancer incidence in Zibo may partly reflect the cumulative lag effect of previous long-term exposure. BaP is a Group I carcinogen that promotes the malignant transformation of bronchial epithelial cells by inducing DNA damage and chronic pulmonary inflammation, representing an important environmental risk factor for lung cancer (32, 33). A large nationwide cohort study showed that PM_2.5_ from industrial sources was significantly associated with an increased risk of lung cancer mortality (HR 1.24, 95% *CI* 1.05–1.45) (34). From 2014 to 2022, although the annual average PM_2.5_ level in Zibo decreased by 8.67% per year, it remained above the national Grade II annual standard of 35 μg/m³ (5), indicating sustained population exposure above regulatory thresholds. A spatiotemporal analysis of lung cancer incidence in Zibo from 2015 to 2019 showed an upward trend, with spatial clustering characterized by “low in the middle, high in the periphery and south,” closely matching the distribution of industrial pollution sources and high PM_2.5_ and PAH areas, suggesting a significant spatial relationship between industrial pollution exposure and lung cancer incidence (4). In addition to PM_2.5_ and PAHs, co-pollutants such as benzene series and heavy metals emitted by petrochemical and metallurgical processes can produce synergistic carcinogenic effects, amplifying the health risks of single pollutants (35). These findings suggest that, for industrial cities such as Zibo, strict regulation of key pollution sources, accelerating the green transformation of petrochemical, ceramic, and metallurgical industries, and controlling emissions at the source are essential. In addition, clean energy promotion should be expanded, environmental monitoring strengthened, and health risk research on PM_2.5_ intensified to improve regional air quality and curb the future lung cancer burden.

The age effect showed that lung cancer risk first increased and then declined with age, and the peak incidence was 60–64 years old for women and 70–74 years old for men, consistent with related studies (36, 37). The post-peak decline may reflect competing risks (e.g., death from cardiovascular or cerebrovascular disease before lung cancer diagnosis) and diagnostic bias (e.g., fewer invasive examinations in the oldest old due to frailty), leading to underestimation of the true incidence at advanced ages (38, 39). The age effect reflects the biological and pathological characteristics of health changes with age (40). Our results showed that lung cancer mortality risk increased with age, consistent with Chinese studies (17, 41). With advancing age, prolonged exposure to environmental carcinogens, together with the natural decline of physiological functions and reduced DNA repair capacity, increases the likelihood of cancer development (42). Lung cancer mortality risk coefficients were positive and significant for men aged ≥60 years and women aged ≥70 years, indicating that these age groups are at high risk. Population aging is a key driver of the rising lung cancer incidence and mortality (43). According to the seventh national census, the population aged ≥60 years in Zibo reached 1.09 million, representing 23.24% of the total. This demographic structure will likely continue to drive up crude incidence and mortality rates and aggravate the future disease burden. Without sustained investment in age-friendly cancer care infrastructure, geriatric oncology services, and targeted screening programs for older adults, the public health impact of lung cancer in Zibo is likely to intensify.

The increasing period effects on lung cancer incidence and mortality may primarily reflect cumulative effect of long-term exposure to various risk factors. Although tobacco control and air quality improvement can reduce lung cancer mortality to some extent, their impact on prognosis is limited, as early-life risk accumulation may have already initiated carcinogenesis, and the upward period effect trend has not been effectively curbed (21). The decreasing trend of lung cancer mortality among women may be related to widespread use of kitchen range hoods, greater health awareness, and active participation in lung cancer screening in recent years. The declining cohort effects with successive birth years indicate a lower lung cancer incidence and mortality risk in later-born generations, possibly due to improvements in medical care, health education, and tobacco control (11). leveraging local medical resources, Zibo has continuously implemented public health interventions, including cancer early detection, early diagnosis, and early treatment programs, health education, tobacco control, and weight management initiatives, which may have further contributed to the declining risk in younger cohorts. However, the stagnation of mortality improvement in cohorts born after 1995 warrants attention and may be influenced by recent increases in smoking prevalence in these groups (44).

Our study has several limitations. First, in the age-period-cohort analysis, the period variable included only three time points (2014, 2019, and 2024), which were separated at 5-year intervals. Given the limited number of time points and the short time span, the model may not adequately capture period effect trends. Second, some relatively young birth cohorts showed considerable uncertainty in risk estimates due to small case numbers, and further validation with future data is needed. Third, specific risk factors such as smoking history, occupational exposure, and indoor oil smoke pollution were not included in this study, so a more comprehensive attribution analysis is not possible. Finally, age-standardized rates were calculated using the 2000 Chinese standard population rather than the world standard population, which limits direct international comparability.

## 5 Conclusion

1. This study elucidated the temporal trends and epidemiological characteristics of lung cancer incidence and mortality in Zibo, and disentangled the independent effects of age, period, and birth cohort on lung cancer risk. These findings provide evidence to guide local lung cancer prevention and control strategies. From 2014 to 2024, the age-standardized incidence rate of lung cancer in Zibo continued to rise, while the mortality rate showed a downward trend. Both incidence and mortality rates were substantially higher in males than in females, but the female incidence rate increased faster, warranting particular attention.
2. Zibo is a typical resource-based old industrial city undergoing industrial transition and faces severe population aging. Unlike many other cities, Zibo faces the unique challenge of combined risks from historical industrial pollution and a rapidly aging population. Persistently high levels of PM_2.5_ exceeding national standards, combined with a high smoking prevalence among males, continue to drive the lung cancer burden in Zibo. These findings of this study therefore provide a valuable reference for developing cancer prevention and control strategies in similar resource-based old industrial cities in China.
3. Given the aging population—23.24% of residents aged ≥60 years, exceeding both national and provincial averages—lung cancer is likely to cause increasing disability-adjusted life years and a growing long-term disease burden. It is therefore essential to strengthen targeted health management and early screening for the key population aged ≥60 years and for male high-risk groups. At the same time, control of key risk factors, including industrial air pollution and tobacco exposure, should be intensified at the source.
4. Early prevention remains the cornerstone of lung cancer control, and early intervention in younger cohorts is key to reducing future mortality and alleviating the socioeconomic burden. Future research should further explore the period and cohort effects on lung cancer and their interplay with social and environmental changes.

## Data Availability

The raw data supporting the conclusions of this article will be made available by the authors, without undue reservation.
